# Supplementary material for: No evidence of male-biased sexual selection in a snake with conventional Darwinian sex roles
Source: R Soc Open Sci. 2020 Oct 7;7(10):201261. doi: 10.1098/rsos.201261 (PMC7657906; doi:10.1098/rsos.201261)
Supplement: SUPPLEMENTAL MATERIALS [file rsos201261supp1.docx]

**Supplemental Materials:**

No Evidence of Male-Biased Sexual Selection in a Snake with Conventional Darwinian Sex Roles

Brenna A. Levine^1,2^, Gordon W. Schuett^2,3^, Rulon W. Clark,^2,4^, Roger A. Repp^5^, Hans-Werner Herrmann^2,6^, and Warren Booth^1,2^

^1^ Department of Biological Science, The University of Tulsa, Tulsa, OK 74104, USA

^2^ The Chiricahua Desert Museum, Rodeo, NM 88056, USA

^3^ Department of Biology and Neuroscience Institute, Georgia State University, Atlanta, GA 30303, USA

^4^ Department of Biology, San Diego State University, San Diego, CA 92182, USA.

^5^ National Optical Astronomy Observatory, Tucson, AZ 85719, USA

^6^ School of Natural Resources and the Environment, University of Arizona, AZ 85721, USA

Corresponding author: Brenna A. Levine (levine.brenna.a@gmail.com)

**Keywords:** Bateman principles, reproductive success, mating system, anisogamy, rattlesnake, *Crotalus atrox*

**1. Bateman gradient analysis with males that did not produce offspring**

Data for male western diamond-backed rattlesnakes (*Crotalus atrox*) with non-zero mating and reproductive success (N = 27; Clark et al., 2014) were analyzed and results reported in the body of the manuscript. However, Clark et al. (2014) also studied 51 males that did not produce offspring during their study. To test for an effect of mating success on reproductive success using all males, including those with zero mating and reproductive success, a generalized linear model (GLM) with a Poisson error distribution was ran. This model included number of years that the male bred as a covariate, and also included an interaction between mating success and number of breeding years. The male *β_SS_* _­_was statistically significant (*β_SS_* = 1.56, *P* < 0.01) when males with zero mating and reproductive success were included in analyses. However, the interaction between mating success and number of breeding years had a significant effect on reproductive success (*P* < 0.01), such that the effect of mating success on reproductive success could not be interpreted without considering this interaction. In other words, a significant effect of mating success on reproductive success depends on the number of years that the individual bred. This result is unsurprising given that individuals that had zero breeding years necessarily also had zero mating success.

**2. Re-analysis of published copperhead data**

To consider the effect of removal of males with zero mating and reproductive success on sexual selection estimates, the data of Levine et al. (2015) were re-analyzed. Levine et al. (2015) analyzed mating and reproductive success for 71 male copperheads (*Agkistrodon contortrix*). Of these, 26 males had non-zero mating and reproductive success.

First, the male *β_SS_* and standardized *β_SS_* were quantified using generalized linear models with mating success as a predictor and reproductive success as the response variable. The number of breeding years for males was not included as a covariate in either model because there were no males that produced offspring in more than one year. For the *β_SS_* GLM, a Poisson error distribution was employed, whereas a Gaussian error distribution was used for the standardized *β_SS_* model. The significance of mating success in each model was assessed using the *drop1{stats}* function in *RStudio*. The female standardized *β_SS_* was also estimated using a linear model with a Gaussian error distribution for subsequent calculation of the female Jones Index.

Next, sex-specific opportunities for sexual selection (*I_s_*) were estimated by dividing sex-specific variance in mating success by sex-specific mean-squared mating success. Similarly, sex-specific opportunities for selection (*I*) were estimated by dividing sex-specific variance in reproductive success by sex-specific mean-squared reproductive success. Male and female *I_s_* and *I* were compared using F-ratio tests.

Finally, the Jones Index (*s’_max_*) was quantified for males and females. To do so, the sex-specific standardized *β_SS_* was multiplied by the square root of sex-specific *I_s_*. Male and female *s’_max_* were statistically compared using an F-ratio test after converting *s’_max_* from standard deviations to variances. Results of these analyses are in the body of the manuscript.

**3. Summary of Clark et al. (2014)**

The present study uses the data of Clark et al. (2014). Clark et al. (2014) studied a single population of *C. atrox* from 2001 – 2010 in the Suizo Mountains (Arizona, USA). Blood samples and SVL measurements were collected from all sampled adults. A subset of adults were also implanted with transmitters and tracked via radio-telemetry at least 2-4 times per month over the course of the study. Female reproductive status of radio-tracked females was determined each year, with gravid females identified. Following parturition and the first sheds of neonates, skin sheds were collected for DNA extraction. Clark et al. (2014) extracted DNA from blood and shed samples, genotyped DNA samples at 27 microsatellite loci, and reconstructed parentage from genotypic data using program COLONY2 (Jones and Wang, 2010). For years in which sires produced offspring but were not sampled, male SVL was corrected based off of growth rates estimated from capture-recapture data and using the male’s SVL for the closest year.

**4. Data Files**

*“atrox_bateman_comparison_4feb20.csv”* – This csv file was used to quantify Bateman gradients for male and female *C. atrox*. The file includes the following information for each snake: snake identifier (“id”), sex (“sex”), total mating success (“mates”), total reproductive success (“offspring”), and the number of years that the individual bred (“years_breeding”).

*“atrox_RRS_StSVL.csv”* – This csv file was used to calculate relative reproductive success and mean-standardized SVL for male *C. atrox* and test for sexual selection and selection on male snout-vent length (SVL). The file includes the following information for each male snake: snake identifier (“Sire”), the year that the snake produced offspring (“Year”), annual reproductive success (“RS”), annual mating success (“MS”), and snout-vent length (“SVL”).

*“opportunities_selection.csv”* – This csv file was used to calculate annual opportunities for sexual selection (*I_s_*) and selection (*I*) for male and female *C. atrox*. The file includes the following information for each snake: snake identifier (“ID”), sex (“Sex”), the year that the individual produced offspring (“Year”), annual reproductive success (“RS”), and annual mating success (“MS”).

*“copperheads.csv”* – This csv file contains the published mating and reproductive success data of Levine et al. (2015) for male and female copperheads (*A. contortrix*), including males that both did and did not produce offspring. We used this file to calculate sexual selection metrics for only those males with non-zero mating and reproductive success. The file includes the following information for each snake: sex (“sex”), mating success (“mates”), and reproductive success (“offspring”).

**5.** Code

All R code are available as supplemental material (atrox_sexual_selection_10Apr20.Rmd). The R code and all files necessary to execute it are also available at https://github.com/brenna-levine/W_Diamondback_Sexual_Selection.

**References**

Clark RW, Schuett GW, Repp RA, Amarello M, Smith CF, Hermann H-W. 2014 Mating systems, reproductive success, and sexual selection in secretive species: a case study of the western diamond-backed rattlesnake, *Crotalus atrox*. *PLOS ONE* **9**, 1–12. (doi:10.1371/journal.pone.0090616)

Jones OR, Wang J. 2010 COLONY: a program for parentage and sibship inference from multilocus genotype data. *Mol. Ecol. Res.* **10:** 551 – 555. (doi:10.1111/j.1755-0998.2009.02787.x)

Levine BA, Smith CF, Schuett GW, Douglas MR, Davis MA, Douglas ME. 2015 Bateman-Trivers in the 21st century: sexual selection in a North American pitviper. *Biol. J. Linn. Soc.* **114**, 436-445. (doi:10.1111/bij.12434)
